# Mapping knowledge landscapes and emerging trends in artificial intelligence for antimicrobial resistance: bibliometric and visualization analysis

**DOI:** 10.3389/fmed.2025.1492709

**Published:** 2025-01-28

**Authors:** Zhongli Wang, Gaopei Zhu, Shixue Li

**Affiliations:** ^1^Centre for Health Management and Policy Research, School of Public Health, Cheeloo College of Medicine, Shandong University, Jinan, China; ^2^NHC Key Lab of Health Economics and Policy Research, Shandong University, Jinan, China; ^3^Department of Biostatistics, School of Public Health, Cheeloo College of Medicine, Shandong University, Jinan, China

**Keywords:** artificial intelligence, antimicrobial resistance, antibiotics, bibliometric analysis, deep learning

## Abstract

**Objective:**

To systematically map the knowledge landscape and development trends in artificial intelligence (AI) applications for antimicrobial resistance (AMR) research through bibliometric analysis, providing evidence-based insights to guide future research directions and inform strategic decision-making in this dynamic field.

**Methods:**

A comprehensive bibliometric analysis was performed using the Web of Science Core Collection database for publications from 2014 to 2024. The analysis integrated multiple bibliometric approaches: VOSviewer for visualization of collaboration networks and research clusters, CiteSpace for temporal evolution analysis, and quantitative analysis of publication metrics. Key bibliometric indicators including co-authorship patterns, keyword co-occurrence, and citation impact were analyzed to delineate research evolution and collaboration patterns in this domain.

**Results:**

A collection of 2,408 publications was analyzed, demonstrating significant annual growth with publications increasing from 4 in 2014 to 549 in 2023 (22.7% of total output). The United States (707), China (581), and India (233) were the leading contributors in international collaborations. The Chinese Academy of Sciences (53), Harvard Medical School (43), and University of California San Diego (26) were identified as top contributing institutions. Citation analysis highlighted two major breakthroughs: AlphaFold’s protein structure prediction (6,811 citations) and deep learning approaches to antibiotic discovery (4,784 citations). Keyword analysis identified six enduring research clusters from 2014 to 2024: sepsis, artificial neural networks, antimicrobial resistance, antimicrobial peptides, drug repurposing, and molecular docking, demonstrating the sustained integration of AI in antimicrobial therapy development. Recent trends show increasing application of AI technologies in traditional approaches, particularly in MALDI-TOF MS for pathogen identification and graph neural networks for large-scale molecular screening.

**Conclusion:**

This bibliometric analysis shows the importance of artificial intelligence in enhancing the progress in the discovery of antimicrobial drugs especially toward the fight against AMR. From enhancing the fast, efficient and predictive performance of drug discovery methods, current AI capabilities have revealed observable potential to be proactive in combating the ever-growing challenge of AMR worldwide. This study serves not only an identification of current trends, but also, and especially, offers a strategic approach to further investigations.

## 1 Introduction

Antimicrobial resistance (AMR), which refers to the ability of microbes (bacteria, viruses, or fungi) to prevent antimicrobials from acting against them, has been recognized as a global public health emergency (1). Additionally, World Health Organization (WHO) concluded in a report that if no more effective interventions are made then AMR is expected to cause 10 million deaths annually by 2050 (2). Antibiotics are top-ranked drugs used in both acute-care hospitals and outpatient clinics, but rational and necessary use remains a challenge; irrational and unnecessary usage increases the emergence of multidrug-resistant (MDR) bacterial pathogens, resulting in higher mortality, longer hospital stays and higher healthcare costs (3, 4). Due to the persistent increase in the incidences of antibiotic resistance (AR) and the emergence of MDR, it is crucial to develop better solution for this. Current research activities have mainly targeted biofilm related strategies, quorum sensing and other factors which contribute to bacterial pathogenicity and models focusing on enhancing the knowledge in the use of antibiotics (5–7).

Over the past few years, artificial intelligence (AI) technology has been applied to medical image understanding, disease diagnosis and treatment, and other areas, leading to realistic advances in medical image analysis, disease prediction, and precise treatment (8). Thus, AI plays a critical role in combating AMR, particularly in the identification and design of antimicrobial agents, drug design and structural optimization, mechanism of action, and molecular exploration (9, 10). Deep learning is utilized to automatically analyze the effects of several chemicals on cell morphology and increase drug accuracy and action (11). Furthermore, it has been utilized to uncover new antibiotic and beta-lactamase inhibitors, improving screening and saving time and money while minimizing development failures (12). In 2024, Wong and his team employed graph neural networks to predict antibiotic activity and cytotoxicity in over 12 million molecules (13), finding the substructures of compounds with strong antibiotic activity but low toxicity. Deep learning was notably effective against Methicillin-resistant *Staphylococcus aureus* (MRSA) and resistant Enterococci, demonstrating its competence and potential.

AI technology is transforming multiple medical fields (14), but its specific roles and efficacy in antimicrobial medication discovery and AMR response methods are remain insufficiently explored. For this reason, bibliometrics fills this research need perfectly. By applying mathematical and statistical techniques to analyze quantitative data from scientific literature, bibliometrics enables the tracking of research trends and outputs (15). It uses indicators including the number of published papers, citation rate, and collaboration map to explain developmental processes and identify hot spots in medical, biological, and engineering sciences (16–18). Moreover, numerous bibliometric studies on AI and medicine have been conducted (19–21), which confirms the widespread usage of this intelligent technology in medicine and provides background and literature for this study.

This bibliometrics study aims to guide policymakers, researchers, and medical professionals in leveraging AI to improve antimicrobial medication development and use. Our study examined the past decade’s literature to uncover technological trends, problems, and future prospects in AI for antimicrobial medications. Additionally, this research also seeks to explore how deep learning technologies help develop novel antibiotics to combat the threat of AMR.

## 2 Materials and methods

### 2.1 Data retrieval

**Figure 1:**
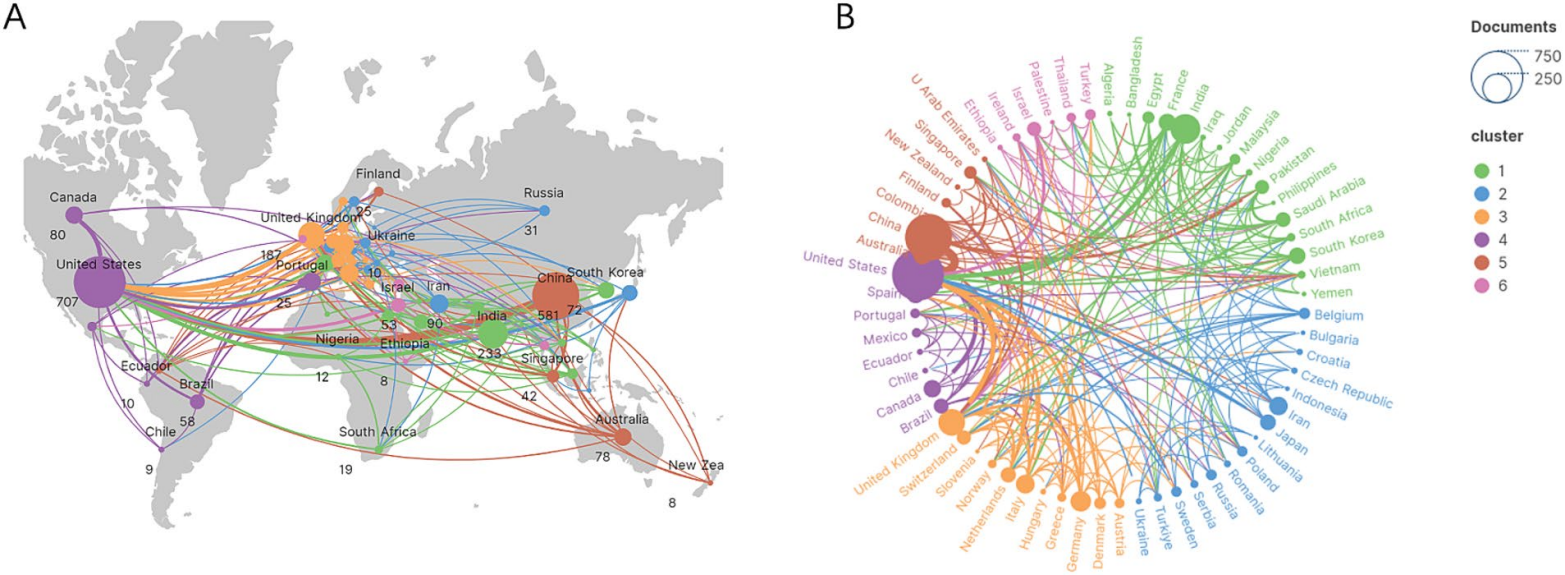
Flowchart for identifying and selecting publications.

The Web of Science Core Collection (WoSCC) is the most well-known and influential database of scientific literature and is the preferred database for bibliometric analysis (22). The WoSCC search was conducted on July 25, 2024. After removing duplicates through automated and manual verification, the final dataset included 2,408 publications from 2014 to 2024. All publications were weighted equally in the analysis to maintain methodological consistency across different research domains. The published period of this study is set between 2014 and 2024. The search terms are as follows: TS = (“anti-infective agent*” OR “anti-bacterial agent*” OR “antimicrobial agent*” OR “antimicrobial use” OR “antibiotic use” OR “antimicrobial residue” OR “antimicrobial resistance” OR “antibiotic resistance” OR antibiotic* OR antifungal* OR antiviral* OR antiparasitic*) AND TS = (“artificial intelligence*” OR “deep learn*” OR “machine learn*” OR “neural network*” OR “compu* intelligent*” OR robot). Only original articles and reviews written in English were included, resulting in a total of 2,408 publications being analyzed in our study (Figure 1).

### 2.2 Data analysis and visualization

Data downloaded from the WoSCC include fully recorded and cited references. We utilized three analysis tools: VOSviewer 1.6.17, CiteSpace 6.3.R3, and Microsoft Excel 2019. Key bibliometric measures include: co-authorship, co-citation, and co-occurrence. For example, co-authorship analysis focused on examining collaborative relationships among authors, nations, or institutions, as indicated by jointly authored papers. The analysis of co-occurrences offers a quantitative approach to identifying relationships among components, while co-citation analysis was employed to evaluate the strength of connections between frequently cited elements.

VOSviewer, developed by Leiden University’s Science and Technology Research Center in the Netherlands, is a tool for creating and exploring network data maps. It offers cluster, superimposed, and density views to evaluate research trends and hotspots (23). In the maps generated by VOSviewer, each node represents an element, with larger nodes indicating more reflections, and broader link widths between nodes indicating a higher level of cooperation. Microsoft Excel was employed to depict the worldwide production and evolutionary trends of relevant papers and to generate charts regarding the rankings of various factors.

CiteSpace is a citation visualization analysis software focused on revealing the potential knowledge contained within the scientific literature (24). Using CiteSpace, we analyzed keyword/reference clustering and timelines. The parameters for CiteSpace included: ① each slice represents a year from 2014 to 2024; ② single node type selection; ③ selection criteria based on the g-index, k = 15; and ④pruning performed using the pathfinder method. The impact factor (IF) and category quartile data were derived from the Journal Citation Reports (JCR) 2023. Scientific researchers, countries, journals, institutions, and journals were evaluated based on the H-index, a composite index measuring the quantity and quality of academic output.

## 3 Results

### 3.1 Analysis of publications

**Figure 2:**
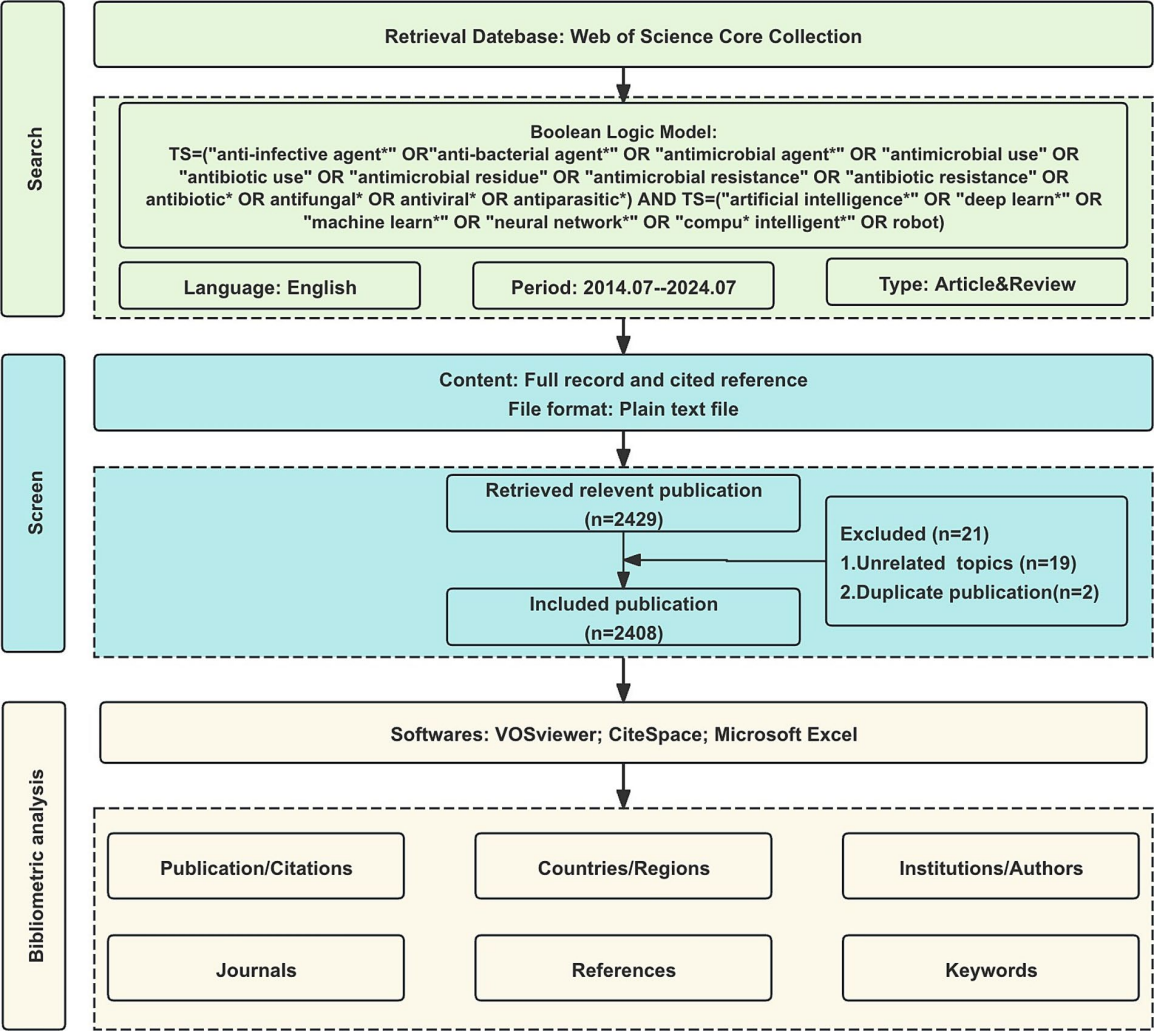
The number of annual publication and the percentage of annual publication.

A collection of 2,408 publications was obtained (key findings are summarized in Supplementary Table S2). As shown in Figure 2, publication output increased moderately from 2014 to 2020, followed by a sharp acceleration that peaked at 549 publications in 2023 (22.7% of total publications). This dramatic increase coincides with significant advances in AI applications for antimicrobial research. The growth pattern can be divided into three distinct phases: (1) an initial steady phase (2014–2019), reflecting the early adoption of AI technologies in antimicrobial research; (2) a transition phase (2019–2021), likely influenced by the emergence of more sophisticated machine learning algorithms and increased computing power; and (3) a rapid expansion phase (2021–2023), marked by breakthrough developments such as AlphaFold’s protein structure predictions and advanced deep learning applications in drug discovery. The steep acceleration post-2020 also correlates with increased funding initiatives and international collaborations focused on AI-driven solutions for antimicrobial drug development.

### 3.2 Analysis of countries/regions

**Figure 3:**
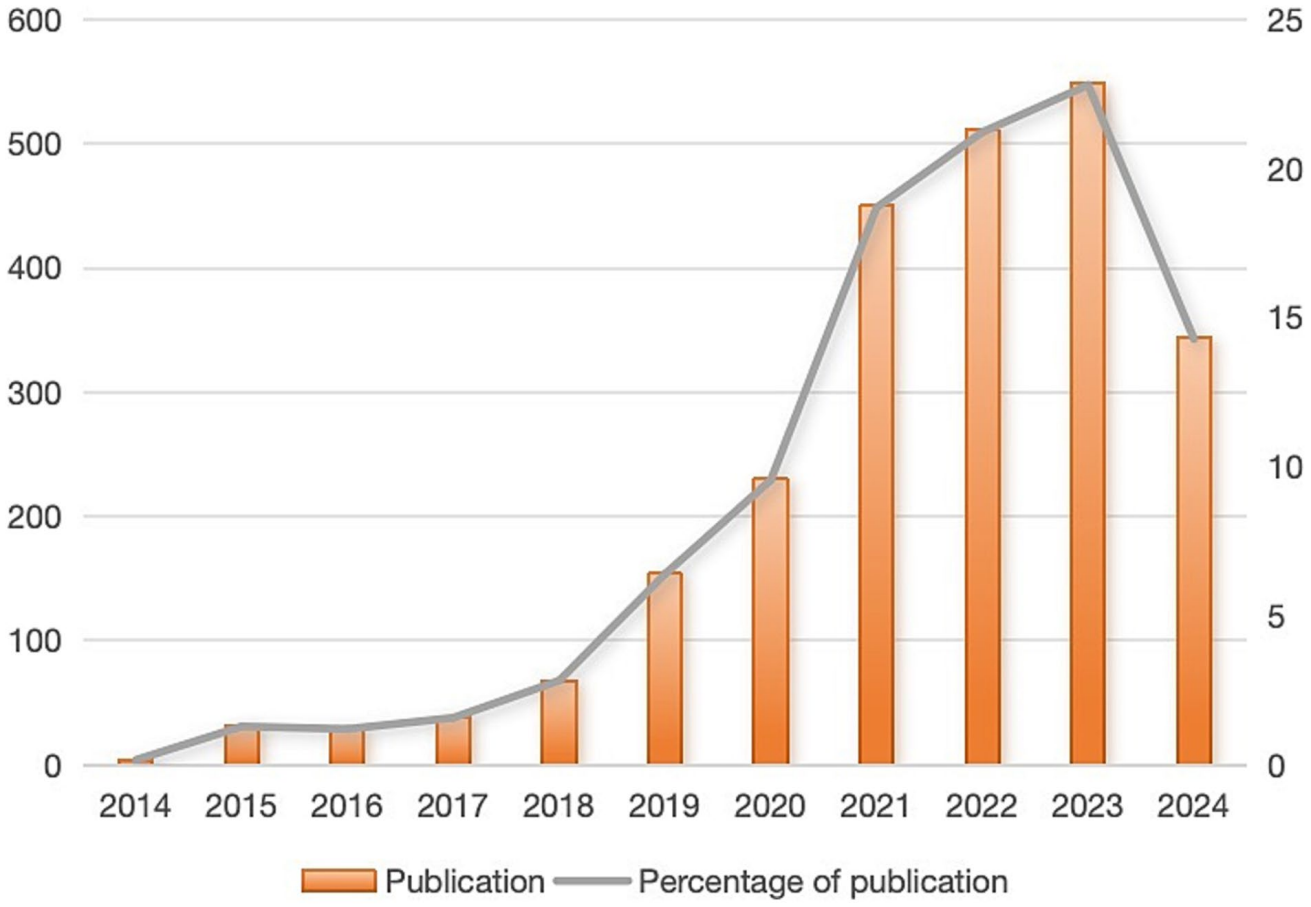
The global research collaboration network. **(A)** Geographic distribution of research collaborations. **(B)** Thematic clusters of global collaborations.

The top 10 countries ranked by their number of publications on the application of AI in antimicrobial agents are highlighted in Table 1. The United States leads with 707 publications, followed by China with 581, and India with 233. The international cooperation network, shown in Figure 3, reveals collaborative relationships among 64 countries. As shown in Figure 3A, the geographic distribution of research collaborations shows strong clustering in North America, East Asia, and Western Europe. Figure 3B further demonstrates that these collaboration networks are particularly dense between the United States and China.

**Table 1:** The top 10 countries/regions in terms of publications.

Rank	Countries	Counts	Citations	TLS
1	United States	707	17,117	511
2	China	581	6,806	273
3	India	233	2,534	157
4	United Kingdom	187	3,717	300
5	Germany	111	1874	193
6	Italy	93	1,131	150
7	Iran	90	1,638	45
8	Spain	89	1,518	146
9	Canada	80	2,945	113
10	Australia	78	1,371	123

### 3.3 Analysis of institutions and authors

**Figure 4:**
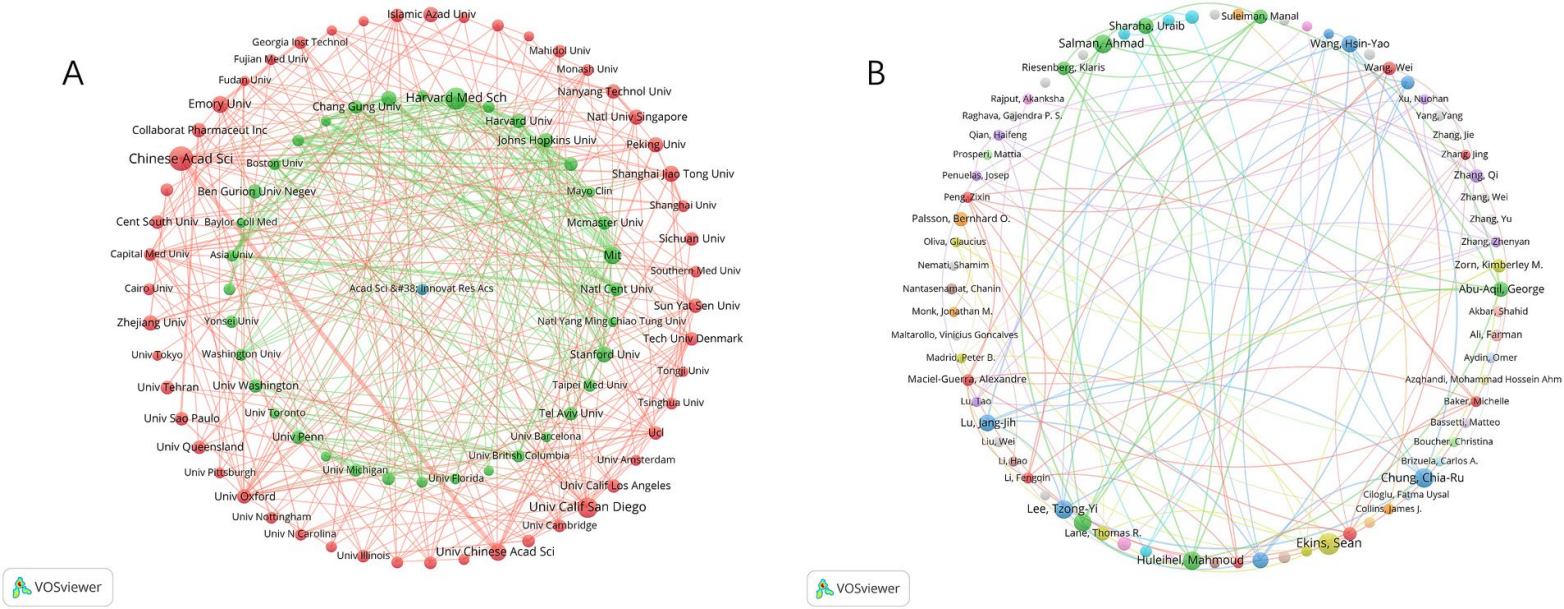
The network map of institutions and authors. **(A)** Institutional collaboration network. **(B)** Author collaboration network.

Leading in publications within this field, the Chinese Academy of Sciences stands out with 53 publications, closely followed by Harvard Medical School and the University of California San Diego, with 43 and 37 publications, respectively, (Table 2). Notably, although Massachusetts Institute of Technology (MIT) ranks fourth with 28 publications, it holds the highest TLS value. This demonstrates that research from the MIT is both extensive and highly interconnected, highlighting its prominent role and influence in global research collaborations. Additionally, Figure 4A reveals the cooperation network among the top 89 institutions by publication volume, identifying two primary clusters. These clusters are distinguished by color, with red representing the cluster centered around the Chinese Academy of Sciences and green for Harvard Medical School. Table 3 lists the top 10 authors by publication volume in this field. Sean Ekins leads with 18 publications, followed by Chia-Ru Chung with 15, and Mahmoud Huleihel with 14. These numbers reflect both their productivity and their impact on the research community. Figure 4B further shows the collaborative networks among these authors.

**Table 2:** The top 10 institutions in terms of publications.

Rank	Institutions	Counts	Citations	TLS
1	Chinese Acad Sci	53	752	701
2	Harvard Med Sch	43	2,761	1885
3	Univ Calif San Diego	37	2,549	1,347
4	Mit	28	2,407	2,251
5	Univ Chinese Acad Sci	26	159	145
6	Emory Univ	25	1,272	1,256
7	Shanghai Jiao Tong Univ	24	256	249
8	Stanford Univ	22	887	867
9	Chinese Univ Hong Kong	21	176	210
10	Peking Univ	21	153	148

**Table 3:** The top 10 authors in terms of publications.

Rank	Author	Count	Country
1	Ekins, Sean	18	United States
2	Chung, Chia-Ru	15	China
3	Huleihel, Mahmoud	14	Israel
4	Lee, Tzong-Yi	14	China
5	Salman, Ahmad	14	Israel
6	Lapidot, Itshak	13	Israel
7	Lu, Jang-Jih	12	China
8	Wang, Hsin-Yao	12	China
9	Horng, Jorng-Tzong	11	China
10	Sharaha, Uraib	11	Israel

### 3.4 Analysis of journals and co-cited journals

**Figure 5:**
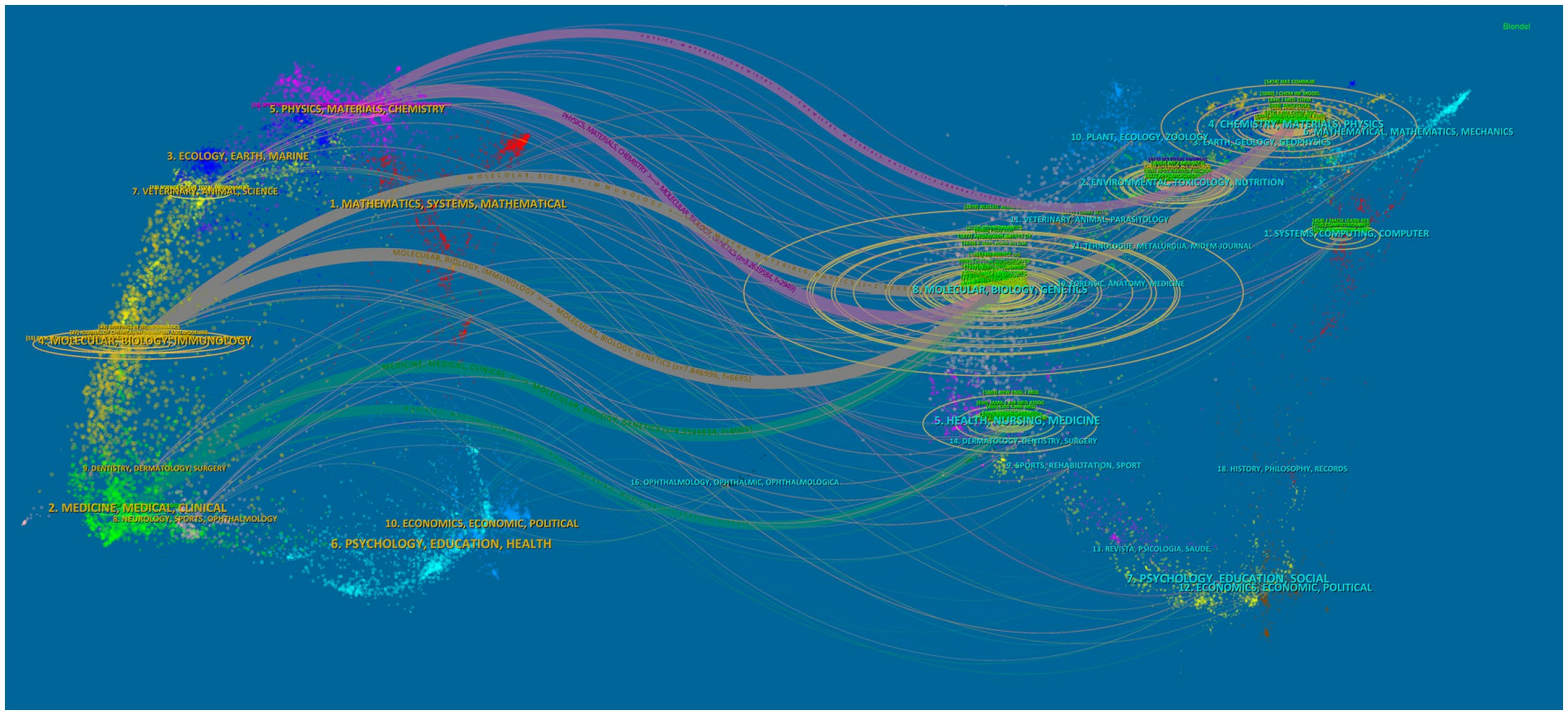
The dual-map overlay of journals represents the topic distribution of academic publications related to AI in antimicrobial resistance research.

Table 4 highlights significant contributions from leading journals in the application of AI to antimicrobial agents. “SCIENTIFIC REPORTS” tops the list with 73 publications, followed by “FRONTIERS IN MICROBIOLOGY” with 67, and “ANTIBIOTICS BASEL” with 50. The publication rankings correlate closely with their citation impacts, with “SCIENTIFIC REPORTS” achieving the highest co-citation count at 4,700. “NATURE COMMUNICATIONS” has the highest IF among the top 10 journals in both publication volume and citations. Furthermore, Figure 5 represents the extensive interconnectedness within scientific research, revealing robust citation flows among fields such as medicine, molecular biology, and health sciences. This comprehensive view highlights the collaborative and multidisciplinary nature of research in AI for antimicrobial applications.

**Table 4:** The top 10 journals and co-cited journals in terms of publications.

Rank	Journals	Count	IF	JCR	Rank	Co-cited Journals	Citations	IF	JCR
1	Scientific Reports	73	3.8	Q1	1	Scientific Reports	526	3.8	Q1
2	Frontiers in Microbiology	67	4.0	Q1	2	Frontiers in Microbiology	487	4.0	Q1
3	Antibiotics Basel	50	4.3	Q1	3	International Journal of Molecular Sciences	387	4.9	Q2
4	Briefings in Bioinformatics	45	6.8	Q1	4	Antibiotics Basel	356	4.3	Q1
5	PLoS One	34	2.9	Q1	5	Science of the Total Environment	269	8.2	Q1
6	Nature Communications	33	14.7	Q1	6	Microorganisms	245	4.1	Q2
7	Molecules	31	4.2	Q2	7	PLoS One	227	2.9	Q1
8	Journal of Chemical Information and Modeling	28	5.6	Q1	8	Molecules	218	4.2	Q2
9	Bioinformatics	24	4.4	Q1	9	Nature Communications	207	14.7	Q1
10	BMC Bioinformatics	24	2.9	Q1	10	Briefings in Bioinformatics	198	6.8	Q1

### 3.5 Analysis of references

**Figure 6:**
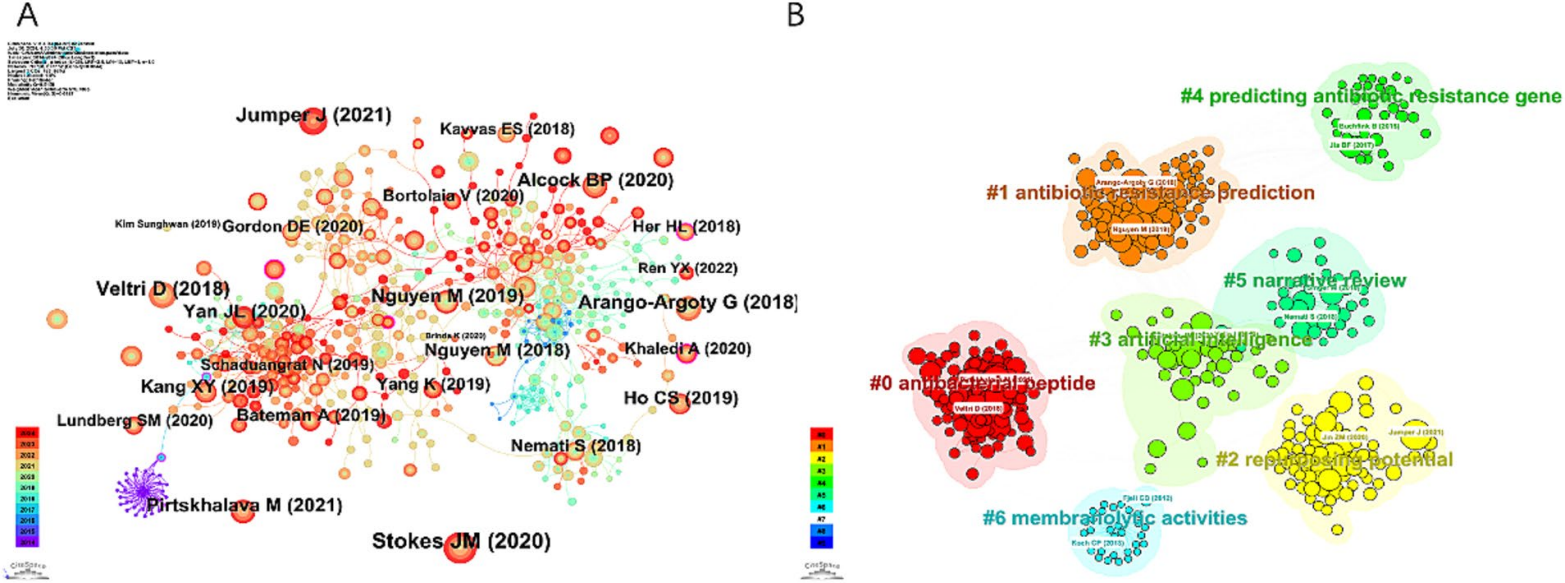
Visualization map of references concerning AI in antimicrobial resistance research. **(A)** Reference co-occurrence network. **(B)** Reference cluster map.

The 10 most frequently co-cited references in research on AI and antimicrobial agents are detailed in Table 5. In 2021, the most frequently co-cited reference was “Highly accurate protein structure prediction with AlphaFold” by Jumper, originally published in Nature (25). This was followed by “A deep learning approach to antibiotic discovery” which appeared in Cell in 2020 (26). The two papers demonstrate significant advancements in the application of AI in antimicrobial agents: AlphaFold’s accurate protein structure predictions facilitate drug discovery and the development of antibiotics, while a deep learning approach has led to the identification of novel antibiotics such as Halicin, offering new avenues to address antibiotic resistance. CiteSpace visualized co-cited references in Figure 6A, and we constructed a network map of the top seven clusters shown in Figure 6B.

**Table 5:** The top 10 co-cited references in terms of publications.

Rank	Co-cited references	Citations	IF	JCR	Centrality
1	Jumper J, 2021, NATURE, V596, P583, DOI 10.1038/s41586-021-03819-2	6,811	50.5	Q1	0.01
2	Stokes JM, 2020, CELL, V180, P688, DOI 10.1016/j.cell.2020.01.021	4,784	98.4	Q1	0.01
3	Alcock BP, 2020, NUCLEIC ACIDS RES, V48, PD517, DOI 10.1093/nar/gkz935	2065	16.6	Q1	0.02
4	Murray CJL, 2022, LANCET, V399, P629, DOI 10.1016/S0140-6736(21)02724-0	989	45.5	Q1	0.02
5	Arango-Argoty G, 2018, MICROBIOME, V6, P0, DOI 10.1186/s40168-018-0401-z	431	13.8	Q1	0.06
6	Veltri D, 2018, BIOINFORMATICS, V34, P2740, DOI 10.1093/bioinformatics/bty179	276	4.4	Q1	0.02
7	Pirtskhalava M, 2021, NUCLEIC ACIDS RES, V49, PD288, DOI 10.1093/nar/gkaa991	243	16.6	Q1	0.01
8	Nguyen M, 2019, J CLIN MICROBIOL, V57, P0, DOI 10.1128/JCM.01260-18	168	6.1	Q1	0.03
9	Yan JL, 2020, MOL THER-NUCL ACIDS, V20, P882, DOI 10.1016/j.omtn.2020.05.006	133	6.5	Q1	0.11
10	Moradigaravand D, 2018, PLOS COMPUT BIOL, V14, P0, DOI 10.1371/journal.pcbi.1006258	109	3.8	Q1	0.01

**Figure 7:**
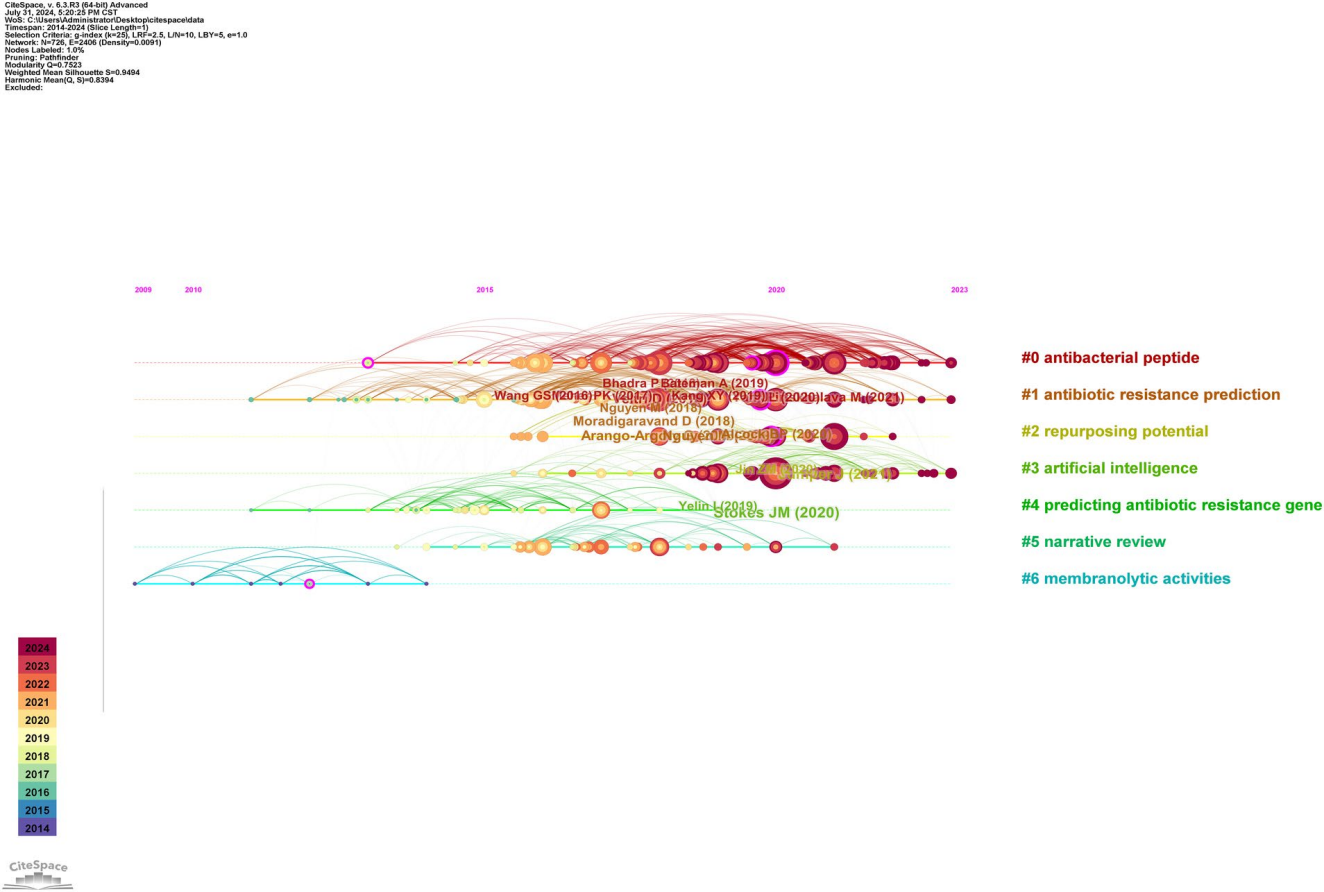
Timeline view of co-cited references by using CiteSpace software.

Clustering reveals dominant themes and trends by grouping similar references. A timeline analysis in Figure 7 further traces the evolution of these trends. Clusters #0 “antibacterial peptide (ABP)” #1 “antibiotic resistance prediction” and #3 “artificial intelligence” have been continuously active research hotspots since their emergence, significantly advancing solutions in global health through innovations in therapy development, drug-resistance management, and medical AI applications.

### 3.6 Analysis of keywords

**Figure 8:**
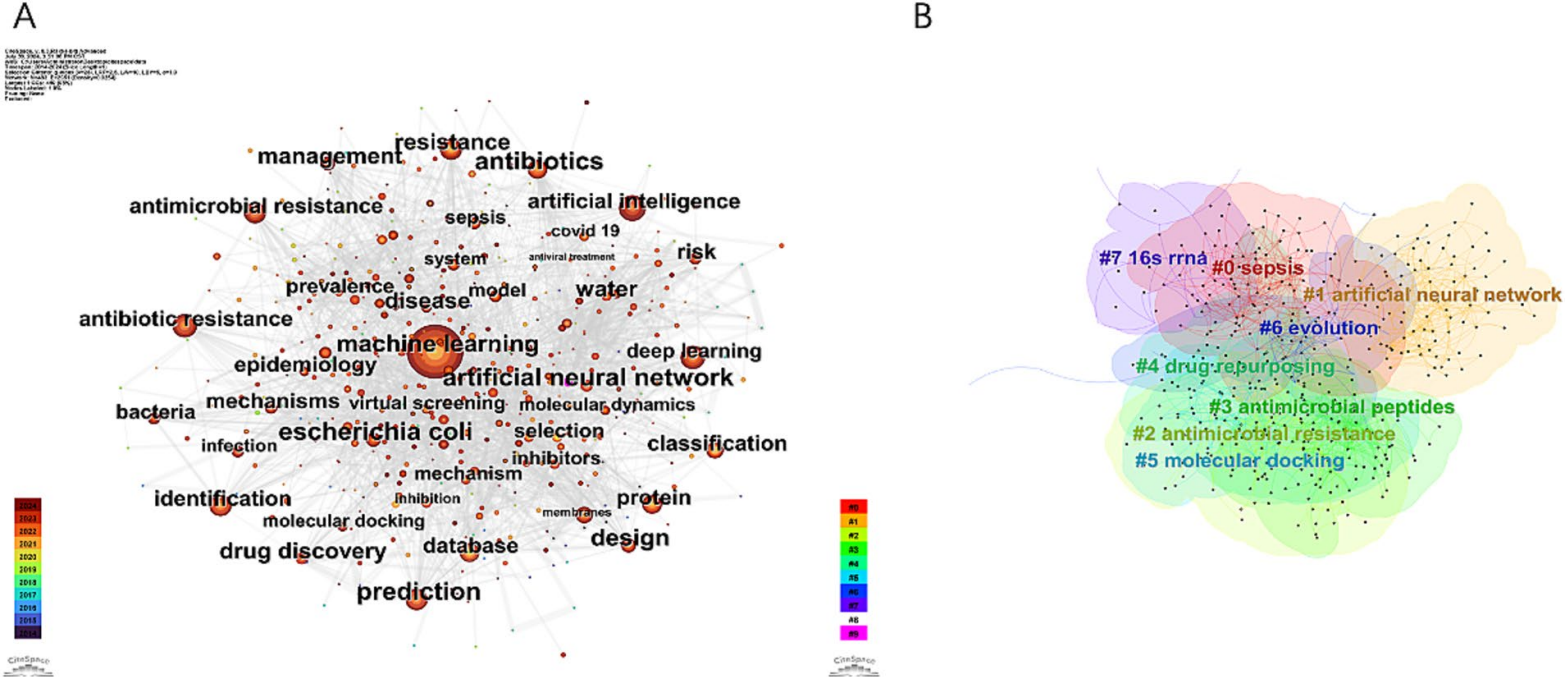
Visualization map of keywords concerning AI in antimicrobial resistance research. **(A)** Keywords co-occurrence network. **(B)** Keywords cluster map.

In the field of AI and antimicrobial agents, a total of 483 keywords were identified, with 33 of these appearing more than 50 times (Figure 8A). Supplementary Table S1 presents the top 20 keywords, which are the most frequently occurring. These keywords are grouped into several thematic categories: AI and data analysis, antibiotic and antimicrobial resistance, microbiology-related terms, and concepts related to biomolecular and drug development. “Machine learning” stands out as the most frequently mentioned keyword, followed closely by “antibiotic resistance” and “prediction” in terms of occurrence.

**Figure 9:**
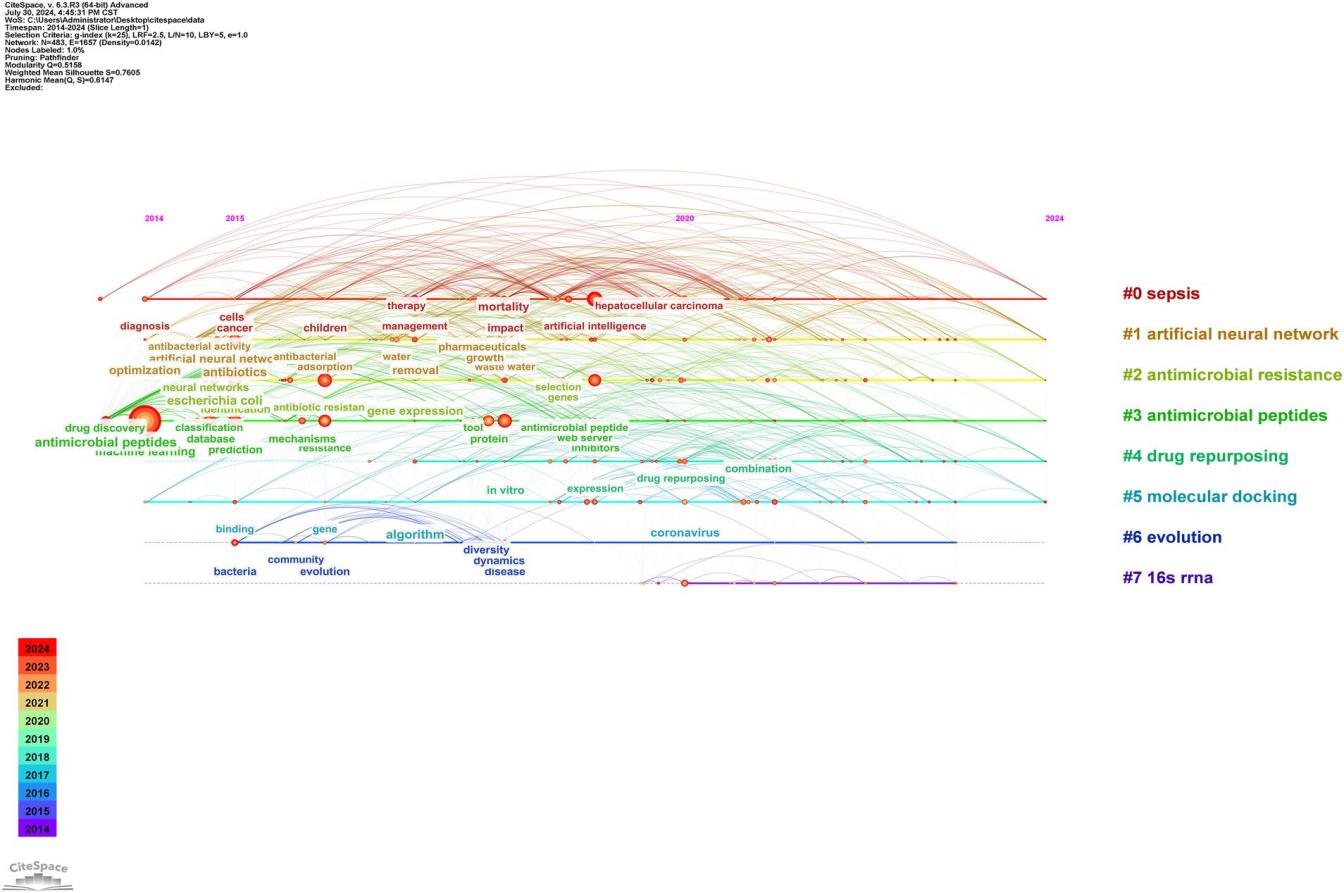
Timeline view of keywords by using CiteSpace software.

Cluster analysis was used to describe the relationships among the themes of keywords, revealing interconnections between AI and antimicrobial agents, as shown in Figure 8B. Timeline analysis further traced the evolution of these themes, as depicted in Figure 9, identifying eight clusters (#0 to #7), highlighting shifts in research focus and emerging trends. The enduring research clusters from 2014 to 2024 in the field of AI and antimicrobial agents—#0 sepsis, #1 artificial neural networks, #2 antimicrobial resistance, #3 antimicrobial peptides, #4 drug repurposing, and #5 molecular docking—highlight the persistent integration of AI in tackling key challenges in antimicrobial therapy.

**Figure 10:**
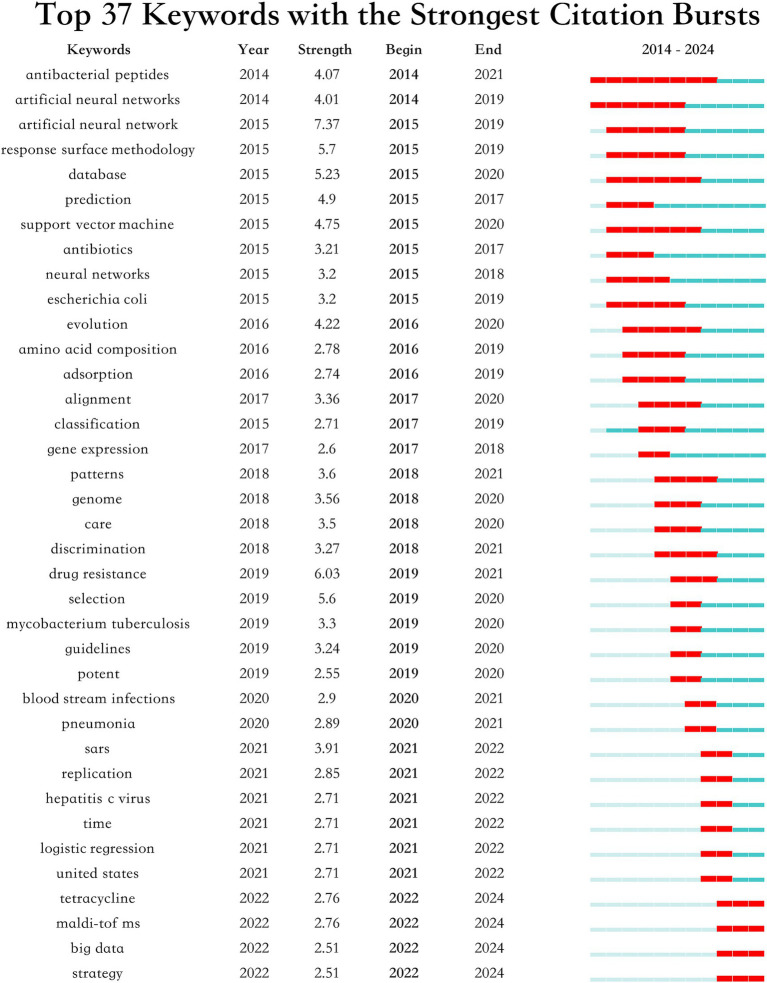
The keywords with the strong citation bursts.

The burst analysis of keywords depicted in Figure 10 underscores significant shifts in research focus and the emergence of new themes within this field. Key trends such as the sustained interest in “ABPs” and “artificial neural networks” from 2014, coupled with the rising concern over “drug resistance” and “*Mycobacterium tuberculosis*” beginning in 2019, reflect crucial developments and policy shifts. Additionally, the analysis highlights growing attention to “tetracycline,” “MALDI-TOF MS,” “big data” and “strategy” indicating a broadening of research scopes and the integration of new technologies and methodologies.

## 4 Discussion

### 4.1 General information

This study analyzed papers over the past decade on AI in antimicrobial drugs, highlighting a marked increase in publications since 2021, peaking in 2023. This trend reflects the urgent need to combat global AMR, emphasizing the vital role of AI. Significant international collaborations, especially among leaders like the United States, China, and India, are essential for fostering technological innovation and interdisciplinary approaches to complex health challenges. The United States leads globally due to its strong research infrastructure and substantial support for new drug development (27). China capitalizes on its extensive medical data and rapid AI innovation (28), while India benefits from its robust IT sector and cost-effective drug development (29).

The contributions of institutions like the Chinese Academy of Sciences, Harvard Medical School, and UC San Diego in AI for AMR are underscored by leading researchers like Sean Ekins, Chia-Ru Chung, and Mahmoud Huleihel. Their prolific work advances the field and highlights the critical role individual researchers play in shaping AMR research (30–33). These institutions stand out due to their robust infrastructure and substantial funding, enabling significant advancements in AMR solutions. Meanwhile, “SCIENTIFIC REPORTS” plays a key role in advancing AI research on antimicrobial drugs, offering a platform for diverse multidisciplinary studies. In contrast, “NATURE COMMUNICATIONS” with its high IF, distinguishes itself by publishing influential, high-quality research that drives global scientific agendas. Additionally, the extensive citation networks connecting disciplines such as medicine and molecular biology further highlight the essential role of interdisciplinary collaboration in addressing AMR challenges.

### 4.2 Research hotspots

The intersection of AI and biomedical research has given rise to significant advancements in the fight against microbial threats. Among these, three studies stand out for their innovative approaches and substantial impact on the field of antimicrobial research.

As the top-ranked co-cited reference (25), the study “Highly accurate protein structure prediction with AlphaFold” by Jumper, published in Nature (2021), marks a pivotal advancement at the juncture of AI and biomedical research. This groundbreaking work on accurately predicting protein structures significantly enhances the field of antimicrobial drug design. By delivering detailed structural predictions of pathogenic proteins, AlphaFold enables the development of targeted antimicrobial therapies that are not only more effective but also less prone to resistance. Additionally, it provides novel insights into ARmechanisms by predicting structural changes in proteins due to mutations, aiding in the design of new drugs to counteract resistance. The rapid and efficient predictions made by AlphaFold facilitate accelerated research and development cycles, essential for keeping pace with swiftly evolving microbial threats. This progress not only advances antimicrobial drug research but also demonstrates the transformative potential of applying advanced computational technologies to biological research, thereby opening new pathways for addressing complex health challenges.

As the second most co-cited reference (26), the paper “A Deep Learning Approach to Antibiotic Discovery” by Stokes, published in Cell (2020), exemplifies the profound impact of computational techniques on antimicrobial drug discovery. This innovative study employed a deep learning model to screen over 107 million compounds efficiently, leading to the discovery of Halicin, a novel antibiotic effective against a broad spectrum of resistant pathogens, including *Mycobacterium tuberculosis*. This research not only illustrates the capacity of computational models to both accelerate and innovate within the antibiotic discovery process but also tackles the pressing issue of antibiotic resistance. It introduces a scalable and novel approach that rejuvenates the antibiotic development pipeline, potentially addressing global health threats.

As the third most co-cited reference (34), “CARD 2020: antibiotic resistome surveillance with the comprehensive antibiotic resistance database” by Alcock, published in Nucleic Acids Research (2020), highlights the essential role of informatics in combating AMR. The Comprehensive Antibiotic Resistance Database (CARD) offers a meticulously curated collection of DNA and protein sequences, along with detection models and bioinformatics tools designed to elucidate the molecular basis of bacterial AMR. This work significantly advances AMR surveillance and analysis capabilities, supporting widespread applications in public health, clinical settings, agriculture, and environmental studies. Through ongoing updates and community-led improvements, CARD not only deepens our understanding of AMR patterns but also strengthens global efforts to effectively manage and reduce antibiotic resistance.

In the field of antimicrobial drugs and AI, significant advancements have been realized through the strategic integration of machine learning technologies. These developments, centered around the key terms “machine learning,” “antibiotic resistance,” and “prediction,” form the backbone of current research efforts aiming to combat the rising challenge of ARefficiently.

In recent years, machine learning has significantly advanced the prediction of antimicrobial drugs and detection of antibiotic resistance. Utilizing deep learning and other machine learning models, researchers have improved diagnostic accuracy. Notably, Stokes (26) used a deep neural network to discover Halicin, a new antibiotic effective against resistant pathogens in mouse models. The evolution of deep learning has also popularized molecular graph representations, enhancing the prediction of antimicrobial activity through graph neural networks (35). Furthermore, Arango-Argoty (36) created DeepARG, a tool for predicting ARgenes using deep learning, and Khaledi (37) applied machine learning to help physicians select targeted antibiotics based on genomic data. AR has become a major challenge in the global public health arena. According to Murray, in 2019, there were 4.95 million deaths associated with bacterial antibiotic resistance, of which 1.27 million deaths were attributed directly to bacterial antibiotic resistance (38). The World Bank estimates that by 2050, ARcould reduce global GDP by up to 3.8% (39). Although the overuse or misuse of antibiotics is a primary driver of resistance, other interconnected factors such as environmental contributors, agricultural practices, and global disparities in antibiotic exposure also exacerbate the prevalence and spread of resistance. For example, the widespread use of antibiotics in agriculture leads to the spread of resistant bacteria through the food chain and environment, impacting human health (40), while urban wastewater treatment plants have become hotspots for antibiotics and resistance genes entering the environment (41). Current antimicrobial prediction models, such as deep learning and random forests, have shown great potential in resistance prediction. However, these models face challenges related to data imbalance, quality control, and missing data (42, 43). Future research should focus on improving model accuracy through data balancing techniques and integrating multiple data sources (such as clinical, genetic, and chemical data) to provide more comprehensive predictions (44).

Recent clinical implementations of AI models in antimicrobial research have shown promising results in several key areas. For example, the clinical validation of deep learning models for antibiotic discovery has shown tangible outcomes. As demonstrated by Stokes, deep learning approaches led to the discovery of novel antibiotics effective against resistant pathogens, particularly against MRSA and resistant Enterococci (26). Furthermore, Wong successfully employed graph neural networks to predict both antibiotic activity and cytotoxicity across over 12 million molecules, identifying compounds with strong antibiotic activity and low toxicity (13). This systematic approach shows great promise for clinical applications. However, challenges remain in widespread clinical adoption, including the need for extensive validation across diverse patient populations and integration with existing hospital information systems.

### 4.3 Future trends

Based on our analysis of publication trends and citation patterns, specific AI models and research priorities emerge as particularly promising for near-term clinical impact. The bibliometric data suggests that graph neural networks, as demonstrated by Wong show particular promise for immediate clinical application due to their proven capability in simultaneously predicting both antibiotic activity and cytotoxicity (13). Additionally, deep learning models for antimicrobial resistance prediction, as shown in Khaledi’s work, demonstrate strong potential for rapid clinical implementation in resistance prediction (37). These approaches are particularly valuable as they can be integrated into existing clinical workflows with minimal disruption.

#### 4.3.1 Application of interdisciplinary methods

There is a problem of integrating artificial neural networks (ANNs) in drug repurposing which can help as one of the solutions to the major global health threat – antibiotic resistance. ANNs are computing systems that mimic the structure and function of the neurons found in biological brains commonly used in the field of Machine Learning as well as Artificial Intelligence. Consisting of several combined layers of neurons such as human brain neurons, ANNs transfer the data through connections and weights (45). Drug repurposing that is also referred to as drug repositioning means using already approved drugs to treat other diseases (46). In this way, ANNs can be used for discovering new potential drugs from the existing libraries and profiles of drug’s resistance, which can be effectively analyzed by using the computational power of ANNs. For example, using mtc-QSAR-EL that contains ensembles of neural networks for virtual screening, researchers have discovered the multi-strain inhibitors for diseases such as tuberculosis and demonstrate the ability to apply these technique to address antibiotic-resistant pathogens (47). Similarly, the work of Tarín-Pelló shows the applicability of TDA for repositioning of FDA-approved drugs for possible effectiveness against *Escherichia coli* and stresses on the flexibility of computation techniques in identifying multifaceted antimicrobial leads (48). Gupta further such AI-based strategies are in the process to become prominent in the improvement of drug design, bettering the reuse of existing drugs and efficiently overcoming the new dimension of AR (49).

These molecular docking techniques have therefore become a very important tool in the analysis of antibiotic resistance among researchers desiring to reduce this vice. For instance, the study done by Das and others used molecular docking and molecular dynamics simulations to study the inhibition of *Staphylococcus aureus* tyrosyl-tRNA synthetase by the newly synthesized glycolipid biosurfactants and thus might be useful in a discovery of new antibacterial agents (50). In the same way, the study by Palazzotti involved the use of supervised molecular dynamics (SuMD) and molecular docking to reveal the mechanisms of interaction between the NorA of *S. aureus* efflux pump and its inhibitors thus opening the way to the development of efflux pump inhibitors to reverse the resistance to antibiotics (51). These examples show a significant role of molecular docking to understand the molecular level principles of drug–target interactions, which can further help in designing and optimizing the drug candidates against AMR.

Mass spectrometry, particularly Matrix-Assisted Laser Desorption/Ionization-Time of Flight Mass Spectrometry (MALDI-TOF MS), has enormous potential that enables the identification of pathogens and their AMR profiles in the shortest time possible. This innovative approach provides a direct and ideal way to analyze the microbial profile and to provide the appropriate antibiotics. For example, Nguyen’s study was able to demonstrate the accuracy of MALDI-TOF MS coupled with AI in predicting AMR in *Pseudomonas aeruginosa* in clinical practice (52). In a similar manner, Wesołowska and Szczuka analyzed the composition of Staphylococci on the skin of healthy animal with help of MALDI-TOF MS, adding more information regarding resistance features even in non-pathogenic flora (53). Further, the study by Teodoro successfully apply MALDI-TOF MS in order to identify the AMR of *Aeromonas* spp. Since the microbial contamination of ready-to-eat foods poses a great concern in food microbiology and public health, the application of this technology is significant (54). Such studies emphasize the significance of MALDI-TOF MS in the modern concepts of the diagnosis of microbial diseases and the fight against AMR.

#### 4.3.2 In-depth analysis of specific areas

Our keyword burst analysis and citation patterns indicate that *Mycobacterium tuberculosis* and sepsis represent critical areas requiring immediate research attention. The surge in tetracycline-related research (starting from 2019) and the persistent focus on drug-resistant pathogens suggest these areas should receive high priority. This aligns with recent WHO priorities and the increasing global burden of these conditions (38, 39). Furthermore, our analysis of emerging trends indicates that the integration of ANNs with drug repurposing strategies shows particular promise for addressing urgent clinical needs in resource-limited settings.

This category of biosensors remains popular to this day because of the idea that ABPs can be used to counteract microbial infections, especially concerning the uncontrolled antibiotic use. AI has the major role in improving the identification and discovery of peptides with high antimicrobial efficacy from the large database of peptides. For example, the work of Yue and his colleagues demonstrates the usage of the ML techniques for the development of a new ABP, IK-16-1 based on human *β*-defensins that demonstrated a broad-spectrum antimicrobial activity, making it potentially perspective for using as a cosmetic preservative (55). In the same way, Yao and the team launched AMPActiPred, a powerful three-step computational tool based on deep forest model to predict the presence and activity level of ABPs, which provides a highly accurate result and user-friendly interface for further study and application (56). Transposing on the possibility of using ABPs to reduce microbial threats, their utilization could as well be adopted for other serious health emergencies, especially sepsis-an extreme response to infection that results to injury of body tissues, organs.

Using ABPs in the fight against microbial threats and the utility of their extension to important health concerns like sepsis that often results from antibiotic-resistant returns our subject to our literature review. Sepsis is a fatal condition that results from an injurious host reaction to an infection thus causing the destruction of host tissues and organs (57). The enhancement of ABPs directed against antibiotic-resistant organisms might be a step forward in the prediction and the handling of sepsis in circumstances in which antibiotics fail. This calls for a more detailed research on the effectiveness and the working of ABPs, especially their applicability as treatment to AR and their potential in intensive care.

Integrating big data analysis enables a comprehensive examination of usage patterns and resistance trends associated with tetracycline, facilitating the development of more effective management strategies. For example, the study by Huang investigates the potential of graphene oxide nanoparticles, enhanced by AI, to remove tetracycline from wastewater, thereby minimizing environmental contamination (58). In a similar vein, Gheytanzadeh and colleagues employ an AI approach to optimize the photocatalytic degradation of tetracycline using metal–organic frameworks (MOFs), underscoring the influence of operational parameters and MOF characteristics on degradation efficiency (59). Additionally, research by Wang assesses the impact of tetracycline on bacterial communities within agricultural soils, revealing substantial alterations in microbial populations and resistance genes, which highlights the ecological implications of antibiotic usage in agricultural settings (60). These studies collectively emphasize the diverse strategies being developed to mitigate tetracycline resistance and environmental pollution, illustrating the pivotal role of innovative technologies and data analysis in advancing solutions for environmental and public health challenges.

### 4.4 Limitation

This study has some limitation, including the use of English only articles from the WoSCC database. Exploring regional databases and non-English publications could offer a more comprehensive view of AI applications in AMR, particularly from regions with high AMR burden but potentially underrepresented in WoSCC. On the positive note, WoSCC is quite expansive but the cost is that great research done in other languages or databases may not be taken into consideration. In addition, the fact that citation delays might lead to underrepresentation of recent high-quality publications could affect the accuracy of trend analyses. However, one cannot deny the fact that this study is appropriate for exploring promising research topics in the development of antimicrobial resistance, revealing some specific research areas that hold the highest potential, as well as the subsequent areas for potential research. The exclusive use of WoSCC is defendable since it along with other limitations offers broad fulltext access and citation analysis, which PubMed or Embase, for instance, does not afford for a bibliometric study.

## 5 Conclusion

The findings from this bibliometric analysis highlight the critical role of AI in addressing AMR. Our comprehensive review spans the years 2014 to 2024, showcasing substantial advancements in AI-driven research within notable institutions and from prominent authors, predominantly from the United States, China, and India. Leading contributors such as the Chinese Academy of Sciences, Harvard Medical School, and MIT have played pivotal roles in international collaborative efforts in this field. This research has significantly advanced the development and optimization of antimicrobial drugs, particularly through groundbreaking applications such as deep learning techniques for predicting antibiotic activity and the accelerated discovery of effective treatments against resistant pathogens. Key references, notably on AlphaFold’s protein structure predictions and AI-driven antibiotic discovery, underscore the transformative impact of these technologies. Additionally, the analysis of keywords and thematic clusters, including “ABPs” and “artificial neural networks” indicates an expanding research scope that not only enhances the drug discovery process but also integrates AI into broader antimicrobial strategies.

## Data Availability

The raw data supporting the conclusions of this article will be made available by the authors, without undue reservation.
