# Supplementary material for: Mapping knowledge landscapes and emerging trends in artificial intelligence for antimicrobial resistance: bibliometric and visualization analysis
Source: Front Med (Lausanne). 2025 Jan 28;12:1492709. doi: 10.3389/fmed.2025.1492709 (PMC11810743; doi:10.3389/fmed.2025.1492709)
Supplement: Supplementary file 1 [file Table_1.DOCX]

| **Rank** | **Keywords** | **Count** | **Centrality** | **Rank** | **Keywords** | **Count** | **Centrality** |
| --- | --- | --- | --- | --- | --- | --- | --- |
| 1 | machine learning | 702 | 0.04 | 11 | database | 100 | 0.01 |
| 2 | antibiotic resistance | 226 | 0.02 | 12 | protein | 93 | 0.02 |
| 3 | prediction | 214 | 0.05 | 13 | antimicrobial peptides | 91 | 0.04 |
| 4 | artificial intelligence | 197 | 0.02 | 14 | classification | 91 | 0.02 |
| 5 | identification | 184 | 0.02 | 15 | artificial neural network | 84 | 0.05 |
| 6 | antimicrobial resistance | 161 | 0.02 | 16 | infection | 83 | 0.01 |
| 7 | deep learning | 146 | 0.01 | 17 | drug discovery | 82 | 0.02 |
| 8 | resistance | 139 | 0.02 | 18 | discovery | 80 | 0.03 |
| 9 | antibiotics | 136 | 0.06 | 19 | design | 79 | 0.04 |
| 10 | escherichia coli | 101 | 0.09 | 20 | bacteria | 79 | 0.01 |
