# Supplementary material for: Mapping knowledge landscapes and emerging trends in artificial intelligence for antimicrobial resistance: bibliometric and visualization analysis
Source: Front Med (Lausanne). 2025 Jan 28;12:1492709. doi: 10.3389/fmed.2025.1492709 (PMC11810743; doi:10.3389/fmed.2025.1492709)
Supplement: Supplementary file 2 [file Table_2.DOCX]

**KEY FINDINGS AT A GLANCE**

Publication Trends:

- Sharp increase in publications from 2021-2023 (549 publications in 2023)
- 22.7% of total publications occurred in 2023 alone

Leading Contributors:

- Countries: USA (707 publications), China (581), India (233)
- Institution: Chinese Academy of Sciences (53 publications)
- Most cited paper: AlphaFold protein structure prediction (6,811 citations)

Research Hotspots:

- Machine learning applications in resistance prediction
- Deep learning for novel antibiotic discovery
- Integration of AI with traditional antimicrobial strategies

Emerging Trends:

- ABPs and artificial neural networks (2014-present)
- Drug resistance and mycobacterium tuberculosis (2019-present)
- MALDI-TOF MS and big data applications (recent surge)
